# Regional gray matter volume is associated with motor imagery performance in children with and without developmental coordination disorder

**DOI:** 10.1093/cercor/bhaf280

**Published:** 2025-10-13

**Authors:** Mugdha Mukherjee, Christian Hyde, Pamela Barhoun, Kaila M Hamilton, Peter G Enticott, Jarrad A G Lum, Karen Caeyenberghs, Nandita Vijayakumar, Jacqueline Williams, Tim Silk, Mervyn Singh, Jessica Waugh, Gayatri Kumar, Ian Fuelscher

**Affiliations:** School of Psychology, Deakin University, 221 Burwood Hwy, Burwood VIC 3125, Australia; School of Psychology, Deakin University, 221 Burwood Hwy, Burwood VIC 3125, Australia; School of Psychology, Deakin University, 221 Burwood Hwy, Burwood VIC 3125, Australia; School of Psychology, Deakin University, 221 Burwood Hwy, Burwood VIC 3125, Australia; School of Psychology, Deakin University, 221 Burwood Hwy, Burwood VIC 3125, Australia; School of Psychology, Deakin University, 221 Burwood Hwy, Burwood VIC 3125, Australia; School of Psychology, Deakin University, 221 Burwood Hwy, Burwood VIC 3125, Australia; School of Psychology, Deakin University, 221 Burwood Hwy, Burwood VIC 3125, Australia; Murdoch Children's Research Institute, 50 Flemington Rd, Parkville VIC 3052, Australia; Institute for Health and Sport, Victoria University, 70/104 Ballarat Rd, Footscray VIC 3011, Australia; School of Psychology, Deakin University, 221 Burwood Hwy, Burwood VIC 3125, Australia; Murdoch Children's Research Institute, 50 Flemington Rd, Parkville VIC 3052, Australia; Cumming School of Medicine, University of Calgary, 3330 Hospital Dr NW, Calgary, AB T2N 2T8, Canada; School of Psychology, Deakin University, 221 Burwood Hwy, Burwood VIC 3125, Australia; School of Psychology, Deakin University, 221 Burwood Hwy, Burwood VIC 3125, Australia; School of Psychology, Deakin University, 221 Burwood Hwy, Burwood VIC 3125, Australia

**Keywords:** childhood, DCD, freesurfer, hand rotation task, morphology

## Abstract

To date, the neurobiological principles that underlie poor motor imagery (MI) performance in children with developmental coordination disorder (DCD) remain poorly understood. To provide new insights into the neuro-structural correlates of MI performance in DCD, this study examined the association between the volume of gray matter regions and MI performance in a sample of 65 children (33 females, 24 children with DCD) aged 6 to 14 yr (mean age = 10.07, SD = 2.64). Implicit MI performance was assessed using a hand laterality judgment task. Regional volumes of frontal-motor, parietal, and cerebellar regions were derived from T1-weighted neuroimaging data. Relative to typically developing children, children with DCD showed less efficient MI performance on the hand laterality judgment task and had smaller cortical volumes in frontal and cerebellar regions. Partial correlations demonstrated that smaller gray matter volumes in frontal and parietal regions were associated with less efficient MI performance in children with and without DCD. These findings provide novel insight into the neurobiological basis of MI performance in children with and without DCD and highlight the possible contribution of gray matter morphological properties to compromised internal models in children with DCD.

## Introduction

Developmental coordination disorder (DCD) is a neurodevelopmental disorder marked by compromised motor skill acquisition and performance in the absence of any known medical or neurological condition (American Psychiatric Association, 2013). Affecting 5% to 6% of school-aged children (Blank et al. 2019), DCD has a considerable impact on everyday movement activities such as using utensils, dressing, toileting, and handwriting (Zwicker et al. 2018; Reynolds et al. 2024). Illustrating the severe impact of DCD, children with DCD experience lower quality of life (Karras et al. 2019), increased rates of mental health difficulties (Omer et al. 2019), psychosocial difficulties (Mancini et al. 2024), and reduced physical health (Philips et al. 2016; Gambra et al. 2024) when compared to their typically developing (TD) peers.

While the etiology of DCD is not fully understood, the internal modeling deficit hypothesis (IMD; Adams et al. 2014) posits that difficulties generating and/or engaging internal action representations may contribute to atypical motor function in DCD (Adams et al. 2014; Scott et al. 2021). Also known as forward models (Miall and Wolpert 1996), internal action representations allow individuals to predict the sensory consequences of their intended actions. These subconscious predictions occur rapidly and facilitate real-time monitoring of the unfolding movement before actual sensory feedback is available (Shadmehr et al. 2010; Ishikawa et al. 2016). In children with DCD, the IMD hypothesis suggests that difficulties generating and/or engaging internal action representations may impact their ability to predict the sensory consequences of intended movements, which may impact their performance on a variety of motor tasks.

In support of the IMD hypothesis, research demonstrates that children with DCD show less efficient performance on motor imagery (MI) tasks (see Barhoun et al. 2019 for a review). MI refers to the process of mentally simulating an action without engaging in overt movement (Parsons 1987). Imagined and overt movements are considered to recruit overlapping neural systems (Hétu et al. 2013; O’Shea and Moran 2017; Hardwick et al. 2018) and show similar temporal and biomechanical characteristics (Kilteni et al. 2018). These similarities between MI and overt movement execution have led to the suggestion that MI tasks may provide insight into the internal models of action that are known to subserve movement (Gabbard 2009; Hurst and Boe 2022).

A commonly used and well-validated measure of MI ability in children is the hand rotation task (HRT; Parsons 1987). During the task, participants are asked to identify the laterality of single-hand stimuli presented at various angular rotations and postural views. The use of MI during the HRT can be inferred from participants’ behavioral profiles on the task since imagined hand rotations have been found to be constrained by the same temporal and biomechanical limitations as physically rotating hands (Butson et al. 2014; Spruijt et al. 2015). That is, children completing the task are faster and more accurate in identifying hand stimuli that are physically comfortable to perform (medial rotations) in comparison to identifying hand stimuli that are physically more challenging to perform (lateral rotations; ter Horst et al. 2010).

Although children with DCD can engage in MI during the HRT (Barhoun et al. 2019), evidence suggests that they perform the HRT slower (Fuelscher et al. 2016; Adams et al. 2017) and with reduced accuracy (Williams et al. 2013; Reynolds et al. 2015) when compared to their TD peers. While this finding has been replicated across several studies (see Barhoun et al. 2019 for a review), research is yet to fully establish the neurobiological mechanisms that underlie less efficient MI performance in DCD. This represents a significant gap in the literature since a better understanding of these mechanisms can provide new insights into the aetiological basis of compromised MI performance in DCD.

From a neurobiological perspective, a growing body of functional MRI research in adults suggests increased neural activation in frontal motor regions (including the inferior, middle, and superior frontal gyri), parietal regions (inferior and superior parietal lobes), and parts of the cerebellum when participants perform the HRT (Hétu et al. 2013; Zapparoli et al. 2014; Hardwick et al. 2018). In support of the view that variations in gray matter morphology within these regions may contribute to less efficient MI performance in DCD, studies have demonstrated morphological differences between children with and without DCD in frontal motor (Langevin et al. 2015; Reynolds et al. 2017; Malik et al. 2024), parietal (Langevin et al. 2015), and cerebellar (Gill et al. 2022) regions.

For example, Reynolds et al. (2017) showed reduced gray matter volume in children with DCD relative to controls in frontal motor areas, including the middle, medial, and superior frontal gyri. Similarly, Gill et al. (2022), demonstrated gray matter volume reductions in parts of the cerebellum in children with DCD relative to children without DCD. Complementing this work, distinct patterns of cortical thinning in frontal and parietal regions in children with DCD were observed by Langevin et al. (2015). In the context of functional MRI studies implicating these regions during MI (Hétu et al. 2013; Zapparoli et al. 2014; Hardwick et al. 2018), this work provides indirect evidence that gray matter morphological properties of these regions may be associated with less efficient MI performance in children with DCD.

To summarize, recent work has provided indirect evidence that variations in gray matter morphology may be associated with less efficient MI performance in children with DCD. However, to the best of our knowledge, no study to date has tested for an association between gray matter morphology and MI performance in children with DCD. This knowledge is valuable as it may enhance our understanding of the neurobiological principles that underlie less efficient MI performance (and atypical motor function) in DCD and ultimately assist in the development and validation of MI training programs that are now commonly adopted to improve motor function in DCD (see Scott et al. 2021 for a review).

The aim of this study was to examine the association between regional gray matter volume and MI performance in a sample of children with and without DCD. MI performance was assessed using the HRT. Gray matter volume of frontal motor (superior and caudal middle frontal, and precentral gyri), parietal (inferior and superior parietal lobes), and cerebellar regions were derived from structural neuroimaging data. These regions were chosen a priori as they are commonly implicated during MI (Hétu et al. 2013; Zapparoli et al. 2014; Hardwick et al. 2018) and previous studies have shown gray matter differences between children with and without DCD in these regions (Langevin et al. 2015; Reynolds et al. 2017; Gill et al. 2022; Malik et al. 2024).

Consistent with earlier accounts, it was hypothesized that children with DCD would show less efficient MI performance and smaller gray matter volumes in frontal motor, parietal, and cerebellar regions relative to children without DCD. It was further hypothesized that individual differences in gray matter volume in these regions would be associated with MI performance in children with and without DCD.

## Materials and methods

### Participants

The sample for this study included 24 children with DCD (17 female; *M*_Age_ = 10.19 yr) and 41 TD children (16 female; *M*_Age_ =  10.01 yr). Following data exclusion (see Supplementary Fig. S1), the final sample used for analysis included 15 children with DCD aged 8 to 14 yr (10 female; *M*_Age_ = 11.74 yr, *SD*_Age_ = 2.06 yr) and 31 TD children aged 6 to 14 yr (15 female; *M*_Age_ = 10.53 yr, *SD*_Age_ = 2.43 yr). Participants were recruited via university advertisements, social media, and clinicians (eg occupational therapists). Parents of participants provided written informed consent, while participants provided their assent. Parents were reimbursed for their time with a gift voucher. Exclusion criteria included a prior diagnosis of an intellectual disability (collected using parent report) and any MRI safety contraindications. All procedures were performed in compliance with relevant laws and institutional guidelines and were approved by the Deakin Human Research Ethics Committee (Ref no. 2019–009; 2018–037).

### Assessment of DCD

Participants were screened against the DSM-5 criteria for DCD (American Psychiatric Association 2013) and assigned to the DCD group in this study if they met the following criteria:

(i) A score at or below the 16th percentile on the short form of the Bruininks–Oseretsky Test of Motor Proficiency 2nd ed. (BOT-2 SF; Bruininks and Bruininks 2005), suggesting motor proficiency significantly below that expected of their age (Criterion A). One participant scored at the 24th percentile on the BOT-2 SF. However, since this participant had an existing diagnosis of DCD from their clinician, and since they met the remaining criteria for DCD in this study, this participant was included in the DCD group; (ii) Evidence that motor difficulties interfered significantly with participants’ abilities to perform daily activities (Criterion B). This criterion was assessed using the Developmental Coordination Disorder Questionnaire (DCD-Q; Wilson et al. 2007). In the absence of Australian norms for DCD-Q score cut-offs, we calculated 95% confidence intervals as detailed in Bianco et al. (2024; see supplementary material). Participants in the DCD group were considered to have met Criterion B if they had a score less than the lower range of the relevant CI (see Supplementary Material); (iii) Evidence that the onset of DCD symptoms occurred during childhood (Criterion C). Participants were deemed to have met this criterion since they were aged 6 to 14 yr; (iv) Evidence that motor skill difficulties were not otherwise attributable to any medical or neurodevelopmental disorders (Criterion D). This criterion was assessed through parent consultations.

Participants in the TD group scored above the 20th percentile on the BOT-2 SF and had not been diagnosed with a developmental disorder (based on parent report).

### Children with attentional difficulties

Children with DCD often present with co-occurring attention-deficit/hyperactivity disorder (ADHD; Goulardins et al. 2017). While participants were not formally assessed for ADHD in this study, we considered the possible impact of attentional difficulties on our results by measuring the severity and frequency of attentional difficulties using the ADHD Rating Scale-IV (ADHD-RS; DuPaul et al. 1998). Sensitivity analyses suggested that our key findings were robust to the possible effect of attentional difficulties (see Supplementary Material). Therefore, we did not covary for attentional difficulties in our final analyses.

### Motor imagery assessment

**Fig. 1:**
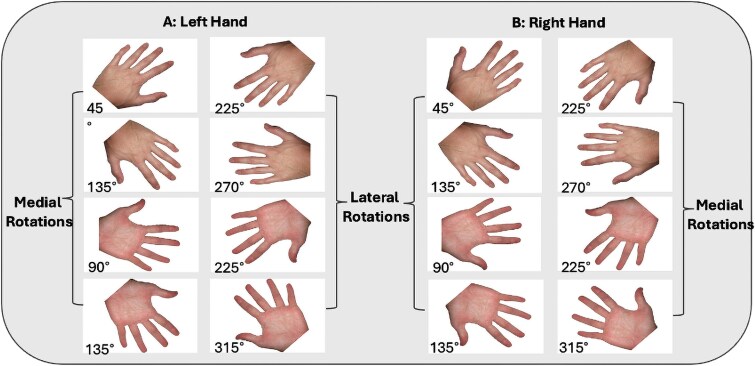
Examples of hand stimuli for left and right hands on the HRT.

The HRT (Parsons 1994) was used to assess MI performance. The task was programmed using E-Prime software (Version 2.0, Psychology Software Tools, Pittsburgh, PA, USA). Single-hand stimuli were presented on a laptop (9 × 8 cm, centered in the middle of the screen), whilst participants were seated upright, with their left index fingers placed on the “D” key of the keyboard (designating left hands) and right index fingers on the “K” key of the keyboard (designating right hands). Participants could see the back of their hands when completing the task. The hand stimuli were presented randomly in either back view (back of the hand facing participants) or palm view (palm of the hand facing participants), in 45° increments between 0° and 360° (Fig. 1). Upon task commencement, participants were asked to determine whether each presented hand stimulus was a left or right hand as quickly and accurately as possible. No specific instructions cueing MI were provided. To discourage the use of a visual matching strategy, participants were instructed to keep their hands on the keyboard during the task. The task took around 4 to 5 min to complete.

The hand stimuli remained on the screen until a response was recorded or until a maximum of 10 s elapsed. Participants completed five practice trials followed by 40 test trials. Response times (RTs) to the nearest millisecond and accuracy were recorded. For statistical analyses, performance was averaged across mirror equivalent angles (e.g. 45° was combined with 315°) to obtain eight trials per angular rotation (0°, 45°, 90°, 135°, 180°). To account for anticipatory responses, trials with RTs less than 250 ms were removed (Kosslyn et al. 1998). This resulted in the removal of 20 trials from 10 participants with DCD and 14 trials from 9 TD participants. As per previous research (Fuelscher et al. 2016; Mukherjee et al. 2024), we applied a minimum accuracy criterion of 60% correct responses for hands presented in back view at 0°/360° to ensure participants were able to differentiate hand laterality at the lowest level of task difficulty. Five participants with DCD and nine participants without DCD did not meet this criterion and were therefore removed from further analyses.

Mean RT and accuracy scores were calculated across angular rotations of 0°, 45°, 90°, 135°, and 180°. We derived mean inverse efficiency scores (IES; Fuelscher et al. 2015; Hyde et al. 2014; Barhoun et al. 2019) for each participant by dividing mean RT by the proportion of correct responses at each angle (lower mean IES values indicate better task performance). As per comparable work (Fuelscher et al. 2015; Hyde et al. 2018; Barhoun et al. 2019), mean IES were used in this study as the preferred performance metric on the HRT as it has been shown to be more sensitive to detect individual differences in HRT performance than RT or accuracy alone (Barhoun et al. 2019). Performance for medially rotated stimuli was calculated as the average response for left hands presented at 45°, 90°, and 135° and right hands presented at 315°, 270°, and 225°. Performance for laterally rotated stimuli was calculated as the average response for left hands presented at 315°, 270°, and 225° and right hands presented at 45°, 90°, and 135°. Two participants with DCD had lateral accuracy scores of 0% and were therefore removed from the analysis.

### Neuroimaging

#### Acquisition

This study was part of a large-scale multimodal MRI study. Participants underwent MR scanning using a Siemens MAGNETOM Prisma 3T scanner (Erlangen, Germany) at the Florey Institute of Neuroscience, Melbourne, Australia. Participants were screened for MRI contraindications (such as implants or metal in the body) and those who were eligible first completed a mock scan. During the actual MR scanning session, participants lay supine on the scanner bed and watched a video of their choice. The scan was administered by professional radiographers who had extensive experience conducting MRI in children. Where the radiographer observed excessive motion, the sequence was repeated if time permitted. High-resolution T1-weighted 3D MPRAGE images were acquired in the sagittal plane, using the following parameters: repetition time TR = 1900 ms, inversion time TI = 900 ms, echo time TE = 2.49 ms, flip angle = 9°, voxel size = 0.9 mm^3^, field of view FoV = 240 mm, 192 contiguous slices with an acquisition time of 4:26 min. The total scanning time was approximately 30 min.

#### Quality assessment

Raw data were assessed both qualitatively and using automated methods. Images were visually inspected for “ringing” and “blurring,” with each rated on a 4-point scale (see Vijayakumar et al. 2021). Images with a rating of 4 on both ringing and blurring (indicating extensive ringing and blurring throughout the brain) were excluded from further analyses. This led to data from two children with DCD and one child in the TD group being removed. Raw images were further processed through MRIQC (Esteban et al. 2017) and assessed for outliers (using the boxplots provided by MRIQC) on intensity nonuniformity (coefficient of joint variation or CJV; Ganzetti et al. 2016), signal-to-noise ratio, ghosting, and blurring (entropy focus criterion or EFC; Atkinson et al. 1997), and bias field (intensity non-uniformity or INU; Tustison et al. 2010). Outlier images were observed for two participants. However, since removing their data from the analysis did not change the general interpretation of findings, these participants were included in the final sample.

#### Image processing

T1-weighted images were processed in FreeSurfer (version 7.4.1; https://surfer.nmr.mgh.harvard.edu) using the “recon-all” command. Processing steps included skull stripping, bias field correction, gray-white matter segmentation, and cortical surface reconstruction. Cortical regions were parcellated using the Desikan–Killiany atlas (Desikan et al. 2006) and subcortical regions were segmented using the default Aseg atlas (Fischl 2012). Processed images were visually inspected for segmentation errors (as described in Vijayakumar et al. 2021) and no scans with consistent and widespread irregularities were identified.

### Regions of interest

**Fig. 2:**
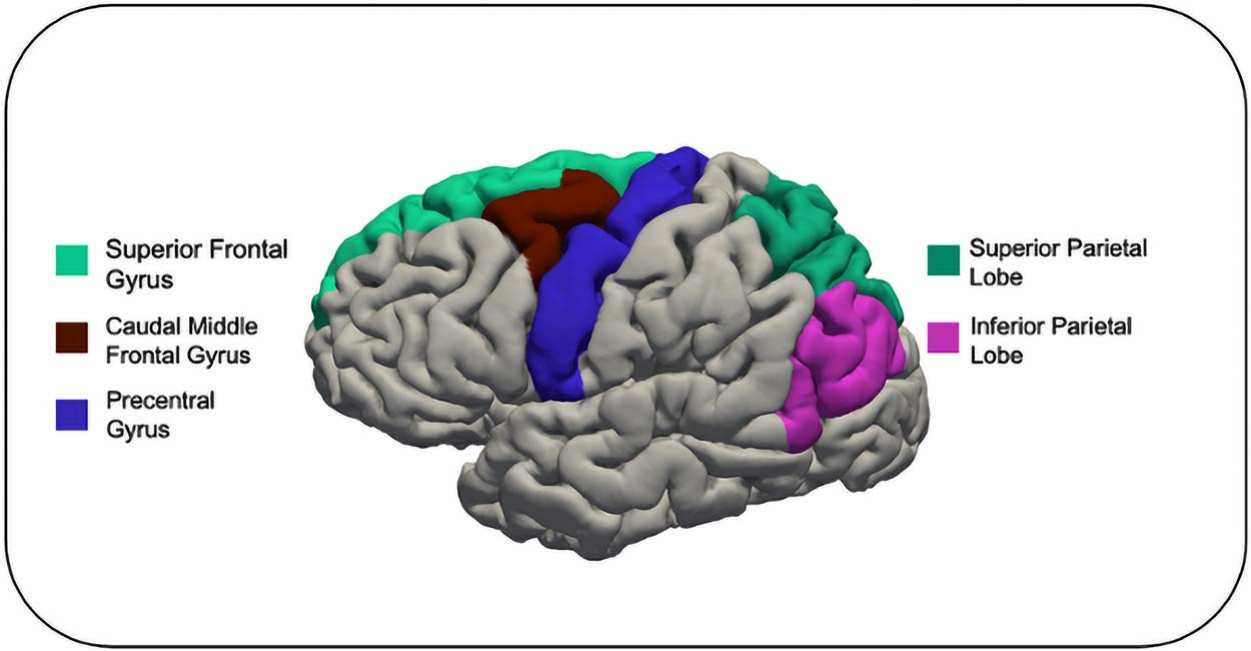
Cortical parcellations of regions of interest included in analyses based on the Desikan–Killiany atlas.

Regional volumes of frontal-motor (left and right hemispheres of the premotor, primary motor, and supplementary motor areas), parietal (left and right hemispheres of the inferior and superior parietal cortices), and cerebellar (left and right cerebellar hemispheres) regions were selected a priori on the basis of previous literature (Hétu et al. 2013; Zapparoli et al. 2014; Hardwick et al. 2018). Volumes of fontal-motor regions were derived using the superior and caudal middle frontal gyrus (comprising parts of the supplementary and premotor areas; Lima et al. 2016; Bhoyroo et al. 2022) and the precentral gyrus (comprising parts of the premotor and primary motor areas; Mayka et al. 2006; Hansen et al. 2022) parcellations from the Desikan–Killiany atlas. Parietal volumes were derived from the Desikan–Killiany atlas using the inferior and superior parietal cortex parcellations. Cerebellar volumes were derived using the left and right cerebellar parcellations from the Aseg atlas. Regions of interest (ROI) are shown in Fig. 2.

### Statistical analyses

#### MI performance

To examine if participants were engaged in a mental rotation strategy during the HRT, we tested for a linear increase in mean IES across angular rotation (0°, 45°, 90°, 135°, 180°) using analysis of variance within each group. To assess if task performance was consistent with the use of a motor-specific mental rotation strategy, and to compare performance between children with and without DCD, a repeated measures analysis of covariance (ANCOVA) was conducted on mean IES with direction of rotation (medial, lateral) and group (TD, DCD) as independent variables. As per comparable research investigating HRT performance in children with DCD (Fuelscher et al. 2015; Reynolds et al. 2015), the use of a motor-specific mental rotation strategy was inferred at a group level due to the modest sample size of the DCD group (see Supplementary Materials). Age and sex were included as covariates since these variables have been shown to be associated with MI performance (Butson et al. 2014; Conson et al. 2020) and since both variables explained a significant amount of variance in MI performance in the present sample.

#### Gray matter volume

Differences in regional gray matter volume between children with and without DCD were assessed using ANCOVA. Age-related differences in global brain volume were accounted for by including total intracranial volume (ICV) as a covariate (Nerland et al. 2022). Sex was initially included as an additional covariate. However, since sex did not explain a significant amount of variance in any of our group difference tests (all *P* > 0.246), and to conserve statistical power, results for group differences in gray matter volume are presented without sex as an additional covariate. Multiple comparisons across ROIs within each hemisphere (*n* = 6) were adjusted using a false discovery rate (FDR) of 0.05 (Benjamini and Hochberg 1995).

#### Association between Gray matter volume and MI performance

Associations between regional gray matter volume and MI performance were examined using Spearman’s rank correlations. A nonparametric approach was adopted since RT-based data are typically positively skewed (Marmolejo et al. 2015). Analyses were conducted across the full sample, given the modest sample size of the DCD group. While our analyses were not sufficiently powered to obtain reliable estimates at a subgroup level, we present separate lines of best fit for the DCD and TD groups to provide a visual representation of the association in each group. Age, sex, and ICV were included as covariates. As per our group comparisons, multiple comparisons across ROIs within each hemisphere (*n* = 6) were adjusted for using a FDR of 0.05.

## Results

### Demographic and clinical characteristics

Demographic and clinical characteristics for the final sample used in the analysis are reported in Table 1. No significant differences in age, sex, or handedness were observed between the groups. Consistent with diagnostic criteria, the DCD group reported lower scores on the BOT-2 and on DCD-Q relative to the TD group. Most participants in our DCD group were female, which is unusual since DCD is more commonly diagnosed in boys (Li et al. 2024). The DCD group scored higher on the ADHD-RS than the TD group. This was expected since children with DCD often present with attentional difficulties (Goulardins et al. 2017).

**Table 1:** Demographic and clinical characteristics.

	**TD**	**DCD**	***P***	**Missing**
*n*	31	15		
Age, yr	10.53 (2.43)	11.74 (2.06)	0.104	ND
Female	15 (48%)	10 (67%)	0.243	ND
Left-handed	6 (19%)	3 (20%)	0.959	ND
BOT2-SF	50.13	12.47	<0.001	ND
DCD-Q	62.35	39.47	<0.001	ND
ADHD-RS	6.30 (5.91)	20.54 (12.46)	<0.001	10 (22%)

Data are presented as mean (SD) or *n*%. Difference tests are based on analysis of variances (continuous variables) or chi-square tests (categorical variables). Two participants in the DCD group had been formally diagnosed by a pediatrician and three participants in the DCD groups had been diagnosed with co-occurring ADHD. No participants in the DCD group had been diagnosed with autism spectrum disorder.

### MI performance

Trend analysis showed a linear increase in mean IES across angular rotation for participants in the TD, *B* = 1755.59, *SE* = 214.81, 95% CI [1330.27, 2180.90], *t*(120) = 8.17, *P* < 0.001, and DCD groups, *B* = 1671.06, *SE* = 423.46, 95% CI [822.76, 2519.36], *t*(56) = 3.95, *P* < 0.001. Descriptive statistics are presented in Table 2.

**Table 2:** Descriptive statistics for MI performance.

	***n***	**Angle**					**Rotation**	
		**0°**	**45°**	**90°**	**135°**	**180°**	**Medial**	**Lateral**
TD	31	2601 (1240)	2840 (1489)	2924 (1182)	3875 (1814)	4859 (2880)	2557 (1106)	4306 (2869)
DCD	15	2914 (1115)	3133 (1625)	3728 (2887)	4161 (1564)	5042 (2231)	3573 (3293)	4558 (2699)

Means and SDs (in brackets) for performance on the HRT. Performance was measured using mean inverse efficiency scores (response time/proportion correct)

**Fig. 3:**
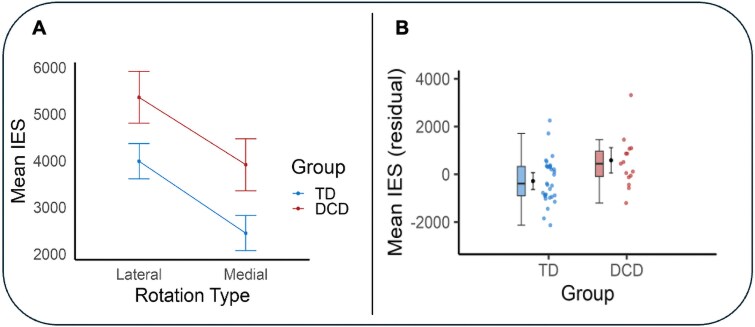
Panel A: Mean IES scores for both groups at each angular rotation. Age and sex were included as covariates. Error bars represent standard errors. Panel B: Mean IES scores for both groups across the HRT. Error bars represent 95% confidence intervals. Outliers for mean IES were considered at a group level and defined as data points further than ±3 SDs from the group mean. No outliers were identified using this approach. IES = mean inverse efficiency scores (response time (ms)/proportion correct).

The repeated measures ANCOVA showed a significant main effect for direction of rotation *F*(1, 42) = 8.30, *P* = 0.006, *η*^2^*_p_* = 0.17. This effect suggests that, averaged across groups, participants showed less efficient performance (higher scores) on lateral rotations compared to medial rotations. The analysis further showed a significant main effect for group *F*(1, 42) = 8.81, *P* = 0.005, *η*^2^*_p_* = 0.17. This effect suggests that, averaged across rotation type, children in the TD group were more efficient on the HRT (lower scores) than children in the DCD group. No significant interaction effect was observed. Descriptive statistics for these analyses are presented in Table 2 and in Fig. 3.

### Gray matter volume

Relative to children in the TD group, children with DCD showed significantly smaller cortical volume in frontal-motor (caudal middle frontal) and cerebellar (left and right cerebellum) regions. These effects are reported in Table 3. Effect sizes for significant group differences were in the medium to large range (Cohen, 1992).

**Table 3:** Descriptive statistics for volumetric measures.

		**TD**	**DCD**	***P***	***p***_***FDR***_	***η*** ^**2**^_**p**_
Left hemisphere						
	Caudal middle frontal gyrus	8.84 (1.39)	7.42 (1.81)	**0.003**	**0.018** ^**a**^	0.19
	Superior frontal gyrus	31.12 (3.75)	29.72 (3.70)	0.285	0.285	0.03
	Precentral gyrus	16.45 (1.72)	15.33 (1.91)	**0.028**	0.056	0.11
	Superior parietal gyrus	17.66 (1.82)	16.76 (1.53)	0.130	0.156	0.05
	Inferior parietal gyrus	17.33 (2.20)	15.90 (3.23)	0.087	0.130	0.07
Right hemisphere						
	Caudal middle frontal gyrus	8.83 (1.60)	7.00 (1.66)	**0.014**	**0.042** ^**a**^	0.13
	Superior frontal gyrus	29.63 (5.05)	27.19 (3.09)	0.070	0.105	0.07
	Precentral gyrus	15.50 (1.83)	14.57 (2.33)	0.175	0.210	0.04
	Superior parietal gyrus	16.53 (2.51)	16.99 (2.39)	0.537	0.537	0.01
	Inferior parietal gyrus	20.67 (3.24)	18.20 (3.89)	**0.033**	0.066	0.10
Cerebellum						
	Left cerebellum	64.14 (6.29)	59.31 (6.53)	**0.011**	**0.033** ^**a**^	0.14
	Right cerebellum	65.00 (5.09)	59.64 (4.97)	**0.006**	**0.036** ^**a**^	0.17

^a^Group comparisons that reach statistical significance following FDR correction (*p_FDR_* < 0.05). Descriptive statistics for volumetric measures (cm^3^) in the TD group (*n* = 31) and in the DCD group (*n* = 15). Data are presented as mean (SD). ICV was included as a covariate. *η*^2^*_p_* = partial eta squared. Values in bold show significant group differences *P* < 0.05.

### Associations between Gray matter volume and MI performance

**Fig. 4:**
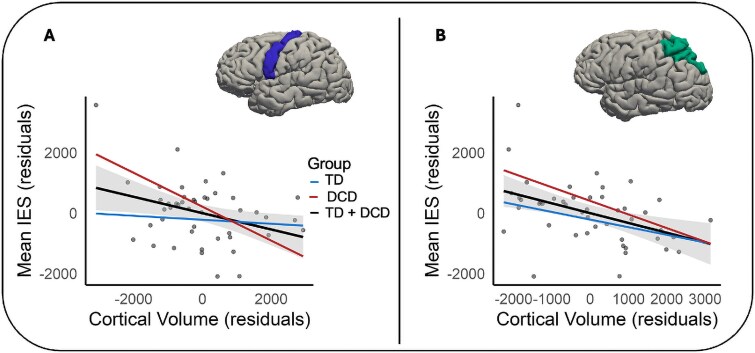
Visual representation of the associations between mean IES and volumetric measures (mm^3^) in children with and without DCD. Panel A: Association in the left precentral gyrus, Panel B: Association in the left superior parietal gyrus. Age, sex, and ICV were included as covariates. IES = mean inverse efficiency score (response time (ms)/proportion correct).

Across the entire sample (children with and without DCD), we observed significant negative associations between mean IES and regional volume of the left precentral gyrus (rho = −0.37, *P* = 0.016, *p*_FDR_ = 0.048) and the left superior parietal gyrus (rho = −0.41, *P* = 0.006, *p*_FDR_ = 0.036). These findings indicated that poorer performance on the HRT task coincided with decreased volumes in these brain regions. These associations are shown in Fig. 4. No further significant associations were observed (see Supplementary Table S1).

## Discussion

This study investigated the association between regional gray matter volume and MI performance in a sample of children with and without DCD. Children with DCD showed less efficient MI performance and were found to have smaller regional gray matter volumes in frontal and cerebellar areas compared to children without DCD. Analyses further demonstrated that smaller gray matter volumes of the left precentral gyrus and the left superior parietal lobe were associated with less efficient MI performance across the entire sample. Results suggest that individual differences in gray matter morphology of the precentral gyrus and the parietal lobe may contribute to less efficient MI performance in children with and without DCD. These findings provide new insights into the neurobiological principals that underlie MI performance in DCD and highlight the possible contribution of gray matter morphological properties to compromised internal models in children with DCD.

### Children with DCD show less efficient MI performance on the HRT

Analyses demonstrated that participants in both the TD and DCD groups were likely engaged in a motor-specific mental rotation strategy during the HRT. Both groups showed a linear increase in mean IES (suggesting less efficient task performance) with increasing stimulus rotation. At the same time, participants were less efficient when responding to biomechanically more difficult (lateral) than easier (medial) rotations, suggesting that task performance was constrained by the biomechanical constraints of real movement. This performance pattern is commonly observed in children with and without DCD (Butson et al. 2014; Reynolds et al. 2015) and indicates the use of a MI strategy during the task.

As hypothesized, children with DCD showed less efficient MI performance (higher mean IES scores) on the HRT compared to children without DCD. This finding is consistent with research demonstrating that children with DCD are slower and less accurate on the HRT relative to their TD peers (Barhoun et al. 2019). Considering that MI performance is thought to provide insight into the ability to generate and/or engage internal models of action (Gabbard 2009; Munzert et al. 2009), our results are compatible with the view that children with DCD in the present sample may have difficulties generating and/or engaging internal models of action (as per the IMD hypothesis; Adams et al. 2014).

### Children with DCD have reduced gray matter volume in sensorimotor regions

As expected, the observed group differences in MI performance between children with and without DCD were paralleled by differences in gray matter volumes of sensorimotor regions. Relative to children in the TD group, children in the DCD group had lower volume in frontal-motor (caudal middle frontal) and cerebellar (left and right cerebellum) regions. These results are largely consistent with past research showing gray matter reductions along the middle frontal gyrus (Reynolds et al. 2017), and the cerebellum (Gill et al. 2022) in children with DCD.

Gray matter differences in childhood are thought to reflect complex interactions between genetic, neurochemical, maturational (eg, synaptogenesis, synaptic pruning), and environmental factors (Lenroot et al. 2009; Brouwer et al. 2015; Norbom et al. 2021). While it is difficult to relate our findings to any given factor, our group comparisons identified several sensorimotor regions that showed smaller gray matter volumes in our sample of children with DCD. Since these regions have been functionally implicated during MI (Hétu et al. 2013; Zapparoli et al. 2014; Hardwick et al. 2018), it is plausible that they may represent neurobiological correlates of less efficient MI performance in DCD. The following section discusses additional evidence in support of this view.

### Gray matter volumes of frontal and parietal regions are associated with MI performance in children with and without DCD

Partial correlation analyses demonstrated that smaller gray matter volume of the left precentral gyrus and the left superior parietal lobe were associated with less efficient MI performance in our sample of children with and without DCD. In the context of the observed MI differences, these results suggest that gray matter structural properties of the left precentral gyrus and the left superior parietal lobe may explain (at least in part) less efficient MI performance in children with and without DCD. No significant associations were observed for the right precentral gyrus and the right superior parietal lobe. This finding is compatible with studies showing that the left cortical hemisphere is often dominant in supporting MI processes in right-handed individuals (eg Hanakawa et al. 2003).

The precentral gyrus (as parcellated by the Desikan-Killiany atlas) comprises parts of the primary motor and premotor cortices (Mayka et al. 2006; Hansen et al. 2022). While the contribution of the primary motor cortex to MI remains debated (Hyde et al. 2017; Bhattacharjee et al. 2021; Barhoun et al. 2022), functional magnetic resonance imaging studies in adults have repeatedly demonstrated that the premotor cortex shows distinct patterns of neural activation when individuals perform MI (Filimon et al. 2015; Pilgramm et al. 2016). Specifically, data suggest that neural signals in the dorsal section of the premotor cortex may play an important role during the imagination of hand actions (Pilgramm et al. 2016).

Action-specific patterns of MI-based neural activation have also been observed in the superior parietal lobe (de Lange et al. 2006; Pilgramm et al. 2016). Based on this work, it has been proposed that MI processes may be supported by a functional network connecting the superior parietal with the dorsolateral premotor cortex (de Lange et al. 2006; Ptak et al. 2017). Our finding that MI performance was associated with structural properties of both the precentral gyrus and the posterior parietal cortex is compatible with this research and provides early evidence that less efficient MI performance in children with and without DCD may be associated with gray matter variations in frontoparietal networks.

While MI performance was associated with gray matter volumes of the precentral gyrus and the superior parietal lobe, the difference tests comparing gray matter volume between the TD and DCD groups in the precentral gyrus fell just short of statistical significance (after adjusting for multiple comparisons) and no significant effect was observed for the superior parietal lobe. However, in the context of earlier work demonstrating select gray matter differences between children with and without DCD in these regions (Langevin et al. 2015; Reynolds et al. 2017) and considering the large effect size observed for the group difference in the precentral gyrus, we propose that our results could reflect the modest sample size of the DCD group rather than a genuine absence of the hypothesized differences.

### Implications and future directions

To our knowledge, this has been the first study to investigate the association between MI performance and regional gray matter morphology in the same sample of children with and without DCD. Results provide new insights into the neurobiological principals that underlie MI performance in DCD and suggest that individual differences in gray matter volume within the precentral gyrus and the superior parietal lobe may be associated with MI performance in children with and without DCD. In doing so, our findings add to a growing body of neuroimaging studies implicating frontoparietal systems in MI broadly, and in DCD.

From a theoretical perspective, our data highlight the possible contribution of gray matter structural properties to compromised internal models in children with DCD (as per the IMD hypothesis; Adams et al. 2014). As noted, the IMD hypothesis suggests that children with DCD may have difficulties generating and/or engaging internal action representations. Since MI is thought to provide insight into the internal action representations that precede movement (Gabbard 2009; O’Shea and Moran 2017), our results are compatible with the view that gray matter variations in frontal and parietal areas may explain (at least in part) compromised internal models in DCD. This knowledge is valuable since it provides new evidence in support of the IMD hypothesis and thus contributes to our understanding of the aetiological basis of DCD.

From a clinical perspective, establishing the neuro-structural systems that subserve MI can assist in the development and objective validation of MI training programs for children with and without DCD. Specifically, our findings highlight the precentral gyrus as a possible neuro-structural region where MI training programs would need to effect change for researchers to conclude that these programs are effective in targeting the underlying mechanisms associated with MI performance. This is of interest to the field since MI training can induce gray matter changes (Lebon et al. 2018) and MI training programs are considered promising for improving movement in children with and without DCD (Behrendt et al. 2021; Scott et al. 2021).

Despite adopting rigorous neuroimaging methods and a well-validated measure of implicit MI, this study is not without limitations. While our design allowed us to detect some of the anticipated group differences and neuro-structural correlates of MI performance, the sample size of our DCD group was modest. This likely reduced the sensitivity of our group comparisons and precluded us from conducting meaningful subgroup analyses in the DCD group. Although separate lines of best fit suggested that the observed associations in the precentral gyrus and the superior parietal lobe were comparable across the DCD and TD groups, future studies should look to replicate our findings in a larger cohort of children with DCD and examine the association between gray matter morphology and MI performance at a subgroup level.

As this has been the first study examining the neuro-structural correlates of MI in children with DCD, and considering our sample size, we opted for an ROI-based approach in our gray matter analyses. However, we acknowledge that the Desikan–Killiany atlas includes spatially extensive ROIs, and that more fine-graded parcellations may provide more sensitive methods for examining subtle gray matter variations and their behavioral correlates. This might also explain why our analyses did not detect some of the hypothesized brain-behavior associations. Adopting finer graded parcellations for examining subtle variations in regional volumes represents an exciting avenue for future research.

## Conclusion

To our knowledge, this has been the first study reporting on the neuro-structural correlates of MI performance in children with and without DCD. Results suggest that smaller gray matter volumes of the left precentral gyrus and the left superior parietal lobe may explain (at least in part) less efficient MI performance in children with and without DCD. These findings add to a growing body of neuroimaging studies implicating frontoparietal systems in MI and provide new insights into the neurobiological basis of MI performance in children with and without DCD. Future research should consider replicating these results in a larger sample of children with DCD and adopt finer graded parcellation algorithms for examining subtle brain-behavior associations in DCD.

## Supplementary Material

Supplementary_Materials_bbaf280

## Data statement

Due to the conditions of our ethics approval, anonymized data from this study is unable to be publicly archived. For readers that are interested in seeking access to the data, please contact the lead author or the relevant local ethics committee. Named individuals will be given access to the data in accordance with ethical procedures governing the reuse of clinical data, including the completion of a formal data sharing agreement and approval from the local ethics committee. Code used for data processing and analysis is publicly available on the FreeSurfer website (https://surfer.nmr.mgh.harvard.edu). Legal copyright restrictions prevent us from publicly archiving the assessment instruments used in this study. This can be obtained from the copyright holders in the cited references.
