# Supplementary material for: Regional gray matter volume is associated with motor imagery performance in children with and without developmental coordination disorder
Source: Cereb Cortex. 2025 Oct 13;35(10):bhaf280. doi: 10.1093/cercor/bhaf280 (PMC12517168; doi:10.1093/cercor/bhaf280)
Supplement: Supplementary_Materials_bbaf280 [file supplementary_materials_bbaf280.docx]

**Regional Grey Matter Volume is Associated with Motor Imagery Performance in Children with and without Developmental Coordination Disorder**

**Supplementary Materials**

Mugdha Mukherjee*^a^, Christian Hyde^a^, Pamela Barhoun^a^, Kaila M. Hamilton^a^, Peter G. Enticott^a^, Jarrad A.G. Lum^a^, Karen Caeyenberghs^a^, Nandita Vijayakumar^a, c^, Jacqueline Williams^b^, Timothy J. Silk^a, c^, Mervyn Singh^d^, Jessica Waugh^a^, Gayatri Kumar^a^, Ian Fuelscher^a^

**Author affiliations**

^a^School of Psychology, Deakin University, Geelong, Victoria, Australia

^b^Institute for Health and Sport, Victoria University, Melbourne, Victoria, Australia

^c^Murdoch Children's Research Institute, Parkville, Australia

^d^Cumming School of Medicine, University of Calgary, Calgary, Canada

**Corresponding Author**

Ms Mugdha Mukherjee, School of Psychology, Deakin University, 221 Burwood Hwy, Burwood VIC 3125, Australia, mmukherj@deakin.edu.au, +61 450 126 658, ORCID: https://orcid.org/0009-0007-5371-2764

**Data Exclusions**

**Supplementary Figure 1.** Sample size flowchart throughout the study. DCD = Developmental Coordination Disorder group, TD = Typically Developing group.

**Descriptive Data for Excluded Participants on the MI Task**

Five participants in the DCD group and nine participants in the TD group were removed from the analysis as they did not meet the minimum accuracy criterion of 60% correct responses for hands presented at the lowest level of task difficulty (Supplementary Figure 1). Comparative descriptive data for these participants is presented in Supplementary Table S1.

|  | | **TD** | | **DCD** | |
| --- | --- | --- | --- | --- | --- |
|  |  | **Included** | **Excluded** | **Included** | **Excluded** |
| **Age** | | 10.53 (2.43) | 8.78 (1.67) | 11.74 (2.06) | 7.70 (3.21) |
| **Left Hemisphere** | |  |  |  |  |
|  | Caudal middle frontal gyrus | 8.84 (1.39) | 8.31 (0.84) | 7.42 (1.81) | 7.81 (1.90) |
|  | Superior frontal gyrus | 31.12 (3.75) | 30.72 (2.72) | 29.72 (3.70) | 26.42 (3.87) |
|  | Precentral gyrus | 16.45 (1.72) | 17.04 (1.21) | 15.33 (1.91) | 15.78 (2.23) |
|  | Superior parietal gyrus | 17.66 (1.82) | 19.11 (1.74) | 16.76 (1.53) | 17.01 (1.90) |
|  | Inferior parietal gyrus | 17.33 (2.20) | 16.97 (1.81) | 15.90 (3.23) | 17.44 (3.67) |
| **Right Hemisphere** | |  |  |  |  |
|  | Caudal middle frontal gyrus | 8.83 (1.60) | 8.99 (1.59) | 7.00 (1.66) | 6.85 (1.28) |
|  | Superior frontal gyrus | 29.63 (5.05) | 30.80 (4.59) | 27.19 (3.09) | 26.68 (3.60) |
|  | Precentral gyrus | 15.50 (1.83) | 15.54 (2.43) | 14.57 (2.33) | 14.46 (2.54) |
|  | Superior parietal gyrus | 16.53 (2.51) | 16.60 (2.21) | 16.99 (2.39) | 16.25 (3.53) |
|  | Inferior parietal gyrus | 20.67 (3.24) | 21.30 (2.11) | 18.20 (3.89) | 19.33 (2.45) |
| **Cerebellum** | |  |  |  |  |
|  | Left cerebellum | 64.14 (6.29) | 63.51 (4.65) | 59.31 (6.53) | 57.03 (6.18) |
|  | Right cerebellum | 65.00 (5.09) | 64.39 (5.20) | 59.64 (4.97) | 58.87 (6.35) |

**Supplementary Table S1.** Descriptive statistics for volumetric measures (cm^3^) in participants included (*n*_TD_ = 31; *n*_DCD_ = 15) and excluded (*n*_TD_ = 9; *n*_DCD_ = 5) from final analyses based on the minimum accuracy criterion on the hand rotation task. Data are presented as mean (SD). TD = Typically Developing group, DCD = Developmental Coordination Disorder group.

**Assessment of DCD (Criterion B)**

Evidence that motor difficulties interfered significantly with participants’ abilities to perform daily activities (Criterion B) was assessed using the Developmental Coordination Disorder Questionnaire (DCD-Q; Wilson et al., 2007). In the absence of Australian norms for DCD-Q score cut-offs, we calculated 95% confidence intervals (Barhoun et al., 2021; Bianco et al., 2024) using data from a larger study from which the present sample was derived. Intervals were calculated for participants aged 5-7 years, 95% CI [59.75 ± 3.48], 8-9 years, 95% CI [64.00 ± 4.44], and 10-14 years, 95% CI [65.05 ± 4.02]. Participants in the DCD group were considered to have met Criterion B if they had a score less than the lower range of the relevant CI.

**Sensitivity Analyses**

**Children with Attentional Difficulties**. We measured the severity and frequency of attentional difficulties using the ADHD Rating Scale-IV (ADHD-RS; DuPaul et al., 1998). Higher raw scores on the ADHD-RS indicate greater severity/frequency of ADHD symptoms. ADHD-RS data were available for 23/31 (74%) children in the TD group and for 13/15 (87%) children in the DCD group. Correlation analyses for this sub-sample (*n* = 36) showed that higher ADHD-RS scores were associated with less efficient MI performance (rho = .41, *p* = .016).To assess the possible impact of attentional difficulties on our key findings, we re-ran the correlation analyses showing significant associations between grey matter organisation and MI performance with ADHD-RS scores as an additional covariate. These analyses suggested that the observed associations remained near identical for the left precentral gyrus (rho_without covariate_ = -.37, rho_with covariate_ = -.32) and the left superior parietal lobe (rho_without covariate_ = -.41, rho_with covariate_ = -.39). Thus, while ADHD-RS scores were associated with MI performance, this had no bearing on our final results. For that reason, and since ADHD-RS data were only available for a subset of participants, we did not covary for attentional difficulties in our analyses.

**MI Strategy.** The use of a motor-specific mental rotation strategy on the HRT can be considered at the individual level by removing participants whose performance is less efficient (lower scores) on medial rotations compared to lateral rotations (for example, see Hyde et al., 2017; Williams et al., 2021) In this study, we considered the use of a motor-specific mental rotation strategy at a group level due to the modest sample size of the DCD group. That is, we included individuals whose performance profile did not conform with the use of a mental rotation strategy in our final sample. This approach is in line with previous work adopting similar sample sizes (Fuelscher et al., 2015; Souto et al., 2020).

To examine the possible impact of this approach on our results, we re-ran the repeated measures ANCOVA examining MI performance after removing participants in the TD (*n* = 1) and DCD (*n* = 5) groups whose performance profile on the HRT was not consistent with that of a MI strategy (these participants were less efficient on medial rotations compared to lateral rotations). As per our analyses for the full sample, we observed a significant main effect for direction of rotation *F*(1, 36) = 23.34, *p* < .001, η^2^_p_ = .39 and a significant main effect for group *F*(1, 36) = 5.76, *p* = .022, η^2^_p_ = .14 This suggests that the rotation effect and group difference test (as reported in the main manuscript) were robust to the removal of participants whose performance profile did not conform with the use of a mental rotation strategy. Accordingly, these participants were included in our final analyses.

Our sensitivity analyses demonstrated a significant interaction effect *F*(1, 36) = 4.74, *p* = .036, η^2^_p_ = .12 which was not observed for the full sample. The interaction effect demonstrated that the difference in HRT performance between children with and without DCD was more pronounced for lateral rotations than for medial rotations. This finding is compatible with the view that children with DCD can perform MI as well as their typically developing peers during biomechanically simple movements but may have difficulties imagining more complex movements. While the observed interaction effect had no bearing on our final analyses in this study, a more detailed consideration of this effect represents an important avenue for future research.

**Effects of Handedness on MI Performance**

To examine the possible effect of stimulus laterality and handedness on response times, we conducted repeated measures ANCOVAs with stimulus laterality (left, right) as the repeated measures factor, handedness (left, right) as the between groups factor, and response time as the dependent variable. Analyses were conducted separately for the DCD and TD groups. Age and sex were included as covariates.

For participants in the DCD group, the repeated measures ANCOVA showed no significant main effect for stimulus laterality, *F*(1, 11) = 0.29, *p* = .599, η^2^_p_ = .03, no significant main effect for handedness, no main effect for participant handedness, *F*(1, 11) = 0.30, *p* = .593, η^2^_p_ = .03, and no significant interaction effect, *F*(1, 11) = 0.01, *p* = .940, η^2^_p_ = .00.

For participants in the TD group, the repeated measures ANCOVA showed no significant main effect for stimulus laterality, *F*(1, 27) = 0.15, *p* = .699, η^2^_p_ = .01, no significant main effect for handedness, *F*(1, 27) = 1.39, *p* = .249, η^2^_p_ = .05, and no significant interaction effect, *F*(1, 27) = 0.04, *p* = .850, η^2^_p_ = .00.

In the context of the non-significant effects observed across these analyses (and considering the small effect sizes), we conclude that neither stimulus laterality nor handedness affected response times on the MI task in this study.

**Association between Grey Matter Volume and MI Performance**

For completeness of results, non-significant associations between MI performance and regional grey matter volumes are presented in Supplementary Table S2.

|  |  | **rho** | ***p*** |
| --- | --- | --- | --- |
| **Left Hemisphere** | |  |  |
|  | Caudal middle frontal | -0.08 | .609 |
|  | Superior frontal | 0.02 | .921 |
|  | Inferior parietal | -0.08 | .591 |
| **Right Hemisphere** | |  |  |
|  | Caudal middle frontal | -0.23 | .146 |
|  | Superior frontal | -0.25 | .107 |
|  | Precentral | -0.22 | .156 |
|  | Inferior parietal | -0.03 | .857 |
|  | Superior parietal | 0.13 | .389 |
| **Cerebellum** | |  |  |
|  | Left cerebellum | 0.01 | .942 |
|  | Right cerebellum | -0.05 | .748 |

**Supplementary Table S2.** Associations between MI performance (mean inverse efficiency scores; IES) and regional grey matter volumes in children with and without DCD (*n* = 46). Age, sex, and ICV were included as covariates.

**References**

Barhoun, P., Fuelscher, I., Do, M., He, J. L., Bekkali, S., Cerins, A., Youssef, G. J., Williams, J., Enticott, P. G., & Hyde, C. (2021). Mental rotation performance in young adults with and without developmental coordination disorder. *Human Movement Science*, *77*, 102787. https://doi.org/10.1016/j.humov.2021.102787

Bianco, K. M., Barhoun, P., Lum, J. A. G., Fuelscher, I., Enticott, P. G., Williams, J., Silk, T. J., Caeyenberghs, K., & Hyde, C. (2024). Atypical procedural learning in children with developmental coordination disorder: A combined behavioral and neuroimaging study. *Brain and Cognition*, *177*, 106160. https://doi.org/10.1016/j.bandc.2024.106160

Butson, M. L., Hyde, C., Steenbergen, B., & Williams, J. (2014). Assessing motor imagery using the hand rotation task: Does performance change across childhood? *Human Movement Science*, *35*, 50–65. https://doi.org/10.1016/j.humov.2014.03.013

Conson, M., De Bellis, F., Baiano, C., Zappullo, I., Raimo, G., Finelli, C., Ruggiero, I., Positano, M., & Trojano, L. (2020). Sex differences in implicit motor imagery: Evidence from the hand laterality task. *Acta Psychologica*, *203*, 103010. https://doi.org/10.1016/j.actpsy.2020.103010

DuPaul, G. J., Power, T. J., Anastopoulos, A. D., & Reid, R. (1998). *ADHD Rating Scale—IV: Checklists, norms, and clinical interpretation* (pp. viii, 79). Guilford Press.

Fuelscher, I., Williams, J., Enticott, P. G., & Hyde, C. (2015). Reduced motor imagery efficiency is associated with online control difficulties in children with probable developmental coordination disorder. *Research in Developmental Disabilities*, *45–46*, 239–252. https://doi.org/10.1016/j.ridd.2015.07.027

Goulardins, J. B., Marques, J. C. B., & De Oliveira, J. A. (2017). Attention Deficit Hyperactivity Disorder and Motor Impairment: A Critical Review. *Perceptual and Motor Skills*, *124*(2), 425–440. https://doi.org/10.1177/0031512517690607

Hyde, C., Fuelscher, I., Lum, J. A. G., Williams, J., He, J., & Enticott, P. G. (2017). Primary Motor Cortex Excitability Is Modulated During the Mental Simulation of Hand Movement. *Journal of the International Neuropsychological Society*, *23*(2), 185–193. https://doi.org/10.1017/S1355617717000029

Souto, D. O., Cruz, T. K. F., Fontes, P. L. B., Batista, R. C., & Haase, V. G. (2020). Motor Imagery Development in Children: Changes in Speed and Accuracy With Increasing Age. *Frontiers in Pediatrics*, *8*. https://www.frontiersin.org/articles/10.3389/fped.2020.00100

Williams, J., Fuelscher, I., & Hyde, C. (2021). Motor imagery in congenital hemiplegia: Impairments are not universal. *Research in Developmental Disabilities*, *114*, 103991. https://doi.org/10.1016/j.ridd.2021.103991
